# Writing implementation research grant proposals: ten key ingredients

**DOI:** 10.1186/1748-5908-7-96

**Published:** 2012-10-12

**Authors:** Enola K Proctor, Byron J Powell, Ana A Baumann, Ashley M Hamilton, Ryan L Santens

**Affiliations:** 1 Center for Mental Health Services Research, George Warren Brown School of Social Work, Washington University in St. Louis, Campus Box 1196, One Brookings Drive, St. Louis, MO, 63130, USA

**Keywords:** Implementation research, Grant writing, Preliminary studies

## Abstract

**Background:**

All investigators seeking funding to conduct implementation research face the challenges of preparing a high-quality proposal and demonstrating their capacity to conduct the proposed study. Applicants need to demonstrate the progressive nature of their research agenda and their ability to build cumulatively upon the literature and their own preliminary studies. Because implementation science is an emerging field involving complex and multilevel processes, many investigators may not feel equipped to write competitive proposals, and this concern is pronounced among early stage implementation researchers.

**Discussion:**

This article addresses the challenges of preparing grant applications that succeed in the emerging field of dissemination and implementation. We summarize ten ingredients that are important in implementation research grants. For each, we provide examples of how preliminary data, background literature, and narrative detail in the application can strengthen the application.

**Summary:**

Every investigator struggles with the challenge of fitting into a page-limited application the research background, methodological detail, and information that can convey the project’s feasibility and likelihood of success. While no application can include a high level of detail about every ingredient, addressing the ten ingredients summarized in this article can help assure reviewers of the significance, feasibility, and impact of the proposed research.

## Background

Investigators seeking funding to conduct implementation research face the challenges of preparing a high-quality proposal and demonstrating their capacity to conduct the proposed study. Researchers need to demonstrate the progressive nature of their research agenda and their ability to build cumulatively upon the literature and their own preliminary studies. Because implementation science is an emerging field involving complex and multilevel processes, most investigators may feel ‘new to the field.’ Furthermore, young investigators may have less preliminary data, and the path to successful proposal writing may seem less clear.

This article identifies ten of the important ingredients in well-crafted implementation proposals; in particular, it addresses how investigators can set the stage for proposed work through pilot data and a well-crafted and rationalized proposed study approach. It addresses questions such as: What preliminary work is important in the grant applications, and how can implementation researchers meet this challenge? How can investigators balance scientific impact with feasibility? Where in an implementation research proposal can investigators demonstrate their capacity to conduct a study as proposed?

### The importance of the question

A significant and innovative research question is the first and primary ingredient in a successful proposal. A competitive implementation research application needs to pursue scientific questions that remain unanswered, questions whose answers advance knowledge of implementation with generalizability beyond a given setting. By definition, implementation research in health focuses on a health condition or disease, healthcare settings, and particular evidence-based interventions and programs with promise of reducing a gap in quality of care. It is conducted in usual care settings with practical quality gaps that stakeholders want to reduce. However, to make a compelling argument for scientific innovation and public health significance, a research grant application must have potential beyond reducing a quality gap and implementing a particular evidence-based healthcare practice. The application must have potential to advance the science of implementation by yielding generalizable knowledge. With only one journal devoted solely to implementation science [1], researchers must be aware of implementation literature that is scattered across a host of discipline-specific journals. Implementation researchers—akin to students with multiple majors—must demonstrate their grounding in implementation science, health diseases, disorders and their treatments, and real-world healthcare delivery.

Although implementation science is often characterized as an emerging field, its bar for scientifically important questions is rising rapidly. Descriptive studies of barriers have dominated implementation science for too long, and the field is urged to ‘move on’ to questions of how and why implementation processes are effective. Accordingly, the Institute of Medicine [2] has identified studies comparing the effectiveness of alternative dissemination and implementation strategies as a top-quartile priority for comparative effectiveness research. But experimental studies testing implementation strategies need to be informed by systematic background research on the contexts and processes of implementation. While investigators must demonstrate their understanding of these complexities, their grant proposals must balance feasibility with scientific impact. This paper addresses the challenges of preparing grant applications that succeed on these fronts. Though this article focuses on U.S. funding sources and grant mechanisms, the principles that are discussed should be relevant to implementation researchers internationally.

### Guidance from grant program announcements

Grant review focuses on the significance of proposed aims, impact and innovation, investigator capacity to conduct the study as proposed, and support for the study hypotheses and research design. The entire application should address these issues. Investigators early in their research careers or new to implementation science often struggle to demonstrate their capacity to conduct the proposed study and the feasibility of the proposed methods. Not all National Institutes of Health (NIH) program announcements require preliminary data. However, those that do are clear that applications must convey investigator training and experience, capacity to conduct the study as proposed, and support for the study hypotheses and research design [3]. The more complex the project, the more important it is to provide evidence of capacity and feasibility [4].

The R01grant mechanism is typically large in scope compared to the R03, R21 and R34^a^. Program announcements for grant mechanisms that are preliminary to R01 studies give important clues as to how to set the stage for an R01 and demonstrate feasibility. Investigator capacity can be demonstrated by describing prior work, experience, and training relevant to the application’s setting, substantive issues, and methodology—drawing on prior employment and research experience. For example, the NIH R03 small grant mechanism is often used to establish the feasibility of procedures, pilot test instruments, and refine data management procedures to be employed in a subsequent R01. The NIH R21 and the R34 mechanisms support the development of new tools or technologies; proof of concept studies; early phases of research that evaluate the feasibility, tolerability, acceptability and safety of novel treatments; demonstrate the feasibility of recruitment protocols; and support the development of assessment protocols and manuals for programs and treatments to be tested in subsequent R01 studies. These exploratory grants do not require extensive background material or preliminary information, but rather serve as sources for gathering data for subsequent R01 studies. These grant program announcements provide a long list of how pre-R01 mechanisms can be used, and no single application can or should provide all the stage-setting work exemplified in these descriptions.

Review criteria, typically available on funding agency web sites or within program announcements, may vary slightly by funding mechanism. However grants are typically reviewed and scored according to such criteria as: significance, approach (feasibility, appropriateness, robustness), impact, innovation, investigator team, and research environment. Table 1 summarizes the ten ingredients, provides a checklist for reviewing applications prior to submission, and ties each ingredient to one or more of the typical grant review criteria.

**Table 1:** Ten key ingredients for implementation research proposals

**Proposal ingredient**	**Key question**	**Review criteria**	**Check (yes/no)**
1. The care gap or quality gap	The proposal has clear evidence that a gap in quality exists?	Significance Impact	
2. The evidence-based treatment to be implemented	Is the evidence for the program, treatment, or set of services to be implemented demonstrated?	Significance Innovation	
3. Conceptual model and theoretical justification	The proposal delineates a clear conceptual framework/theory/ model that informs the design and variables being tested?	Approach Innovation	
4. Stakeholder priorities, engagement in change	Is there a clear engagement process of the stakeholders in place?	Significance Impact Approach Environment	
5. Setting’s readiness to adopt new services/treatments/programs	Is there clear information that reflects the setting’s readiness, capacity, or appetite for change, specifically around adoption of the proposed evidence-based treatment?	Impact Approach Environment	
6. Implementation strategy/process	Are the strategies to implement the intervention clearly defined, and justified conceptually?	Significance Impact Innovation	
7. Team experience with the setting, treatment, implementation process	Does the proposal detail the team’s experience with the study setting, the treatment whose implementation is being studied, and implementation processes?	Approach Investigator team	
8. Feasibility of proposed research design and methods	Does the methods section contain as much detail as possible, as well as lay out possible choice junctures and contingencies, should methods not work as planned?	Approach Investigator team	
9. Measurement and analysis section	Does the proposal clarify the key constructs to be measured, corresponding to the overarching conceptual model or theory?	Approach Investigator team	
	Is a measurement plan clear for each construct?		
	Does the analysis section demonstrate how relationships between constructs will be tested?		
10. Policy/funding environment; leverage or support for sustaining change	Does the proposal address how the implementation initiative aligns with policy trends?	Impact Significance	

## Discussion

### Approach

The literature does not provide a ‘. . . a comprehensive, prescriptive, and robust-yet practical-model to help…researchers understand (the) factors need to be considered and addressed’ in an R01 study [5]. Therefore we examined a variety of sources to identify recommendations and examples of background work that can strengthen implementation research proposals. This paper reflects our team’s experience with early career implementation researchers, specifically through training programs in implementation science and our work to provide technical assistance in implementation research through our university’s Clinical and Translational Science Award CTSA program. We also studied grant program announcements, notably the R03, R21, R18, and R01 program announcements in implementation science [6-9]. We studied how successful implementation research R01 grant applications ‘set the stage’ for the proposed study in various sections of the proposal. We conducted a literature search using combinations of the following key words: ‘implementation research,’ ‘implementation studies,’ ‘preliminary studies,’ ‘preliminary data,’ ‘pilot studies,’ ‘pilot data,’ ‘pilot,’ ‘implementation stages,’ ‘implementation phases,’ and ‘feasibility.’ We also drew on published studies describing the introduction and testing of implementation strategies and those that characterize key elements and phases of implementation research [10,11].

From these reviews, we identified ten ingredients that are important in all implementation research grants: the gap between usual care and evidence-based care; the background of the evidence-based treatment to be implemented, its empirical base, and requisites; the theoretical framework for implementation and explicit theoretical justification for the choice of implementation strategies; information about stakeholders’ (providers, consumers, policymakers) treatment priorities; the setting’s (and providers’) readiness to adopt new treatments; the implementation strategies planned or considered in order to implement evidence-based care; the study team’s experience with the setting, treatment, or implementation process and the research environment; the feasibility and requisites of the proposed methods; the measurement and analysis of study variables; and the health delivery setting’s policy/funding environment, leverage or support for sustaining change.

Given the sparse literature on the importance of preliminary studies for implementation science grant applications, we ‘vetted’ our list of grant application components with a convenience sample of experts. Ultimately, nine experts responded to our request, including six members of the *Implementation Science* editorial board. We asked the experts to rate the importance of each of the ten elements, rating them as ‘1: Very important to address this is the application,’ ‘2: Helpful but not necessary to the application,’ or ‘3: Not very important to address’ within the context of demonstrating investigator capacity and study feasibility. Respondents were also asked whether there are any additional factors that were not listed.

While all the ten ingredients below were considered important for a successful application, several experts noted that their importance varies according to the aims of the application. For example, one expert affirmed the importance of the settings’ readiness to change, but noted that it may not be crucial to address in a given proposal: ‘the setting’s readiness may be unimportant to establish or report prior to the study, because the study purpose may be to establish an answer to this question.’ However, another maintained, ‘in a good grant application, you have to dot all the ‘I’s’ and cross all the ‘T’s.’ I consider all these important.’ One expert noted that applications might need to argue the importance of implementation research itself, including the importance of closing or reducing gaps in the quality of care. This was viewed as particularly important when the study section to review the grant may not understand or appreciate implementation research. In these cases, it may be important to define and differentiate implementation research from other types of clinical and health services research. For example, it may be useful to situate one’s proposal within the Institute of Medicine’s ‘prevention research cycle,’ which demonstrates the progression from pre-intervention, efficacy, and effectiveness research to dissemination and implementation studies that focus on the adoption, sustainability, and scale-up of interventions [12]. It may also be important to convey that implementation research is very complex, necessitating the use of multiple methods, a high degree of stakeholder involvement, and a fair amount of flexibility in order to ensure that implementers will be able to respond appropriately to unforeseen barriers.

### Ten key ingredients of a competitive implementation research grant application

As emphasized at the beginning of this article, the essential ingredient in a successful implementation science proposal is a research question that is innovative and, when answered, can advance the field of implementation science. Assuming that an important question has been established to potential reviewers, we propose that the following ten ingredients can help investigators demonstrate their capacity to conduct the study and to demonstrate the feasibility of completing the study as proposed. For each ingredient, we provide examples of how preliminary data, background literature, and narrative detail in the application can strengthen the application.

### The care gap, or quality gap, addressed in the application

The primary rationale for all implementation efforts, and thus a key driver in implementation science, is discovering how to reduce gaps in healthcare access, quality, or, from a public health perspective, reducing the gap between Healthy People 2020 [13] goals and current health status. Accordingly, implementation research proposals should provide clear evidence that gaps exists and that there is room for improvement and impact through the proposed implementation effort. This is a primary way of demonstrating the public health significance of the proposed work.

Gaps in the quality of programs, services, and healthcare can be measured and documented at the population-, organization-, and provider-levels [14]. Several kinds of preliminary data can demonstrate the quality gap to be reduced through the proposed implementation effort. For example, investigators can emphasize the burden of disease through data that reflect its morbidity, mortality, quality of life, and cost [14]. An implementation research grant should cite service system research that demonstrates unmet need [15], the wide variation in the use of evidence-based treatments in usual care [16-19], or the association between the burden of disease and variations in the use of guidelines [20]. Investigators can also document that few providers adopt evidence-based treatments [21,22], that evidence-based treatments or programs have limited reach [23], or that penetration [24] into a system of care can be addressed by the implementation study. Regardless of the specific approach to documenting a quality gap, investigators should use rigorous methods and involve all relevant stakeholders [14]. In fact, stakeholders can demonstrate their involvement and endorse quality gaps through letters of support attesting to the lack of evidence-based services in usual care.

### The evidence-based treatment to be implemented

A second key ingredient in implementation research proposals is the evidence-based program, treatment, policies, or set of services whose implementation will be studied in the proposed research [25-27]. The research ‘pipeline’ [28-30] contains many effective programs and treatments in a backlog, waiting to be implemented. Moreover, many health settings experience a huge demand for better care. An appropriate evidence-based treatment contributes to the project’s public health significance and practical impact, presuming of course that it will be studied in a way that contributes to implementation science.

Implementation research proposals must demonstrate that the evidence-based service is ready for implementation. The strength of the empirical evidence for a given guideline or treatment [31,32], a key part of ‘readiness,’ can be demonstrated in a variety of ways; in some fields, specific thresholds must be met before an intervention is deemed ‘evidence-based’ or ‘empirically-supported’ [33-35]. For example, Chambless *et al.*[35] suggest that interventions should demonstrate efficacy by being shown to be superior to placebos or to another treatment in at least two between group design experiments; or by showing efficacy in a large series of single case design experiments. Further, Chambless *et al.*[35] note that the experiments must have been conducted with treatment manuals, the characteristics of the samples must have been clearly specified, and the effects must have been demonstrated by at least two different investigators or investigative teams.

The strength of evidence for a given treatment can also be classified using the Cochrane EPOC’s criteria for levels of evidence, which considers randomized controlled trials, controlled clinical trials, time series designs, and controlled before-and-after studies as appropriate [36]. Researchers who come to implementation research as effectiveness researchers or as program or treatment developers are well positioned, because they can point to their prior research as part of their own background work. Other researchers can establish readiness for implementation by reviewing evidence for the treatment or program as part of the background literature review, preferably relying on well-conducted systematic reviews and meta-analyses of randomized-controlled trials (if available). At a minimum, ‘evaluability assessment’ [37] can help reflect what changes or improvements are needed to optimize effectiveness given the context of the implementation effort.

### Conceptual model and theoretical justification

Any research striving for generalizable knowledge should be guided by and propose to test conceptual frameworks, models, and theories [38]. Yet, theory has been drastically underutilized and underspecified in implementation research [38-40]. For example, in a review of 235 implementation studies, less than 25% of the studies employed theory in any way, and only 6% were explicitly theory-based [39]. While translating theory into research design is not an easy task [36], the absence of theory in implementation research has limited our ability to specify key contextual variables and to identify the precise mechanisms by which implementation strategies exert their effects.

McDonald *et al.*[41] present a useful hierarchy of theories and models, which serves to organize the different levels of theory and specify the ways in which they can be useful in implementation research. They differentiate between conceptual models, frameworks, and systems, which are used to represent global ideas about a phenomenon and theory, which is an ‘organized, heuristic, coherent, and systematic set of statements related to significant questions that are communicated in a meaningful whole’ [41]. Within the realm of theory, they differentiate between grand or macro theories (*e.g.*, Rogers’ Diffusion of Innovations theory [26]), mid-range theories (*e.g.*, transtheoretical model of change [42]), and micro-theories (*e.g.*, feedback intervention theory [43]). Though models, frameworks, and systems are generally at a higher level of abstraction than theories, it is important to note that the level of abstraction varies both between and within the categories of the hierarchy. The thoughtful integration of both conceptual models and theories can substantially strengthen an application.

Conceptual models, frameworks, and systems can play a critical role in anchoring a research study theoretically by portraying the key variables and relationships to be tested. Even studies that address only a subset of variables within a conceptual model need to be framed conceptually, so that reviewers perceive the larger context (and body of literature) that a particular study proposes to inform. Given the confusion surrounding definitions and terminology within the still-evolving field of dissemination and implementation [44,45], grant proposals need to employ consistent language, clear definitions for constructs, and the most valid and reliable measures for the constructs that correspond to the guiding conceptual framework or theoretical model. Proposal writers should be cautioned that the theory or conceptual model used to frame the study must be used within the application. A mere mention will not suffice. A conceptual model can help frame study questions and hypotheses, anchor the background literature, clarify the constructs to be measured, and illustrate the relationships to be evaluated or tested. The application must also spell out how potential findings will inform the theory or model.

Numerous models and frameworks can inform implementation research. For example, Glasgow *et al.*[23] RE-AIM framework can inform evaluation efforts in the area of implementation science. Similarly, Proctor *et al.*[46] have proposed a model that informs evaluation by differentiating implementation, service system, and clinical outcomes, and identifying a range of implementation outcomes that can be assessed [24]. Damschroder *et al.*’s [10] Consolidated Framework for Implementation Research identifies five domains that are critical to successful implementation: intervention characteristics (evidentiary support, relative advantage, adaptability, trialability, and complexity); the outer setting (patient needs and resources, organizational connectedness, peer pressure, external policy and incentives); the inner setting (structural characteristics, networks and communications, culture, climate, readiness for implementation); the characteristics of the individuals involved (knowledge, self-efficacy, stage of change, identification with organization, etc.); and the process of implementation (planning, engaging, executing, reflecting, evaluating). Others have published stage or phase models of implementation. For example, the Department of Veteran Affairs’ QUERI initiative [47] specifies a four-phase model spanning pilot projects, small clinical trials, regional implementation, and implementation on the national scale; and Aarons, Hurlburt and Horwitz [48] developed a four phase model of exploration, adoption/preparation, active implementation, and sustainment. Magnabosco [49] delineates between pre-implementation, initial implementation, and sustainability planning phases.

McDonald *et al.*[41] note that grand theories are similar to conceptual models, and that they generally represent theories of change. They differentiate between classical models of change that emphasize natural or passive change processes, such as Rogers’ diffusion of innovations theory [26], and planned models of change that specify central elements of active implementation efforts. Investigators may find it more helpful to draw from mid-range theories because they discuss the mechanisms of change at various levels of the implementation context [26]. For example, social psychological theories, organizational theories, cognitive psychology theories, educational theories, and a host of others may be relevant to the proposed project. While conceptual models are useful in framing a study theoretically and providing a ‘big picture’ of the hypothesized relationships between variables, mid-range theories can be more helpful in justifying the selection of specific implementation strategies specifying the mechanisms by which they may exert their effects. Given the different roles that theory can play in implementation research, investigators would be wise to consider relevant theories at multiple levels of the theoretical hierarchy when preparing their proposals. It is far beyond the scope of this article to review conceptual models and theories in detail; however, several authors have produced invaluable syntheses of conceptual models and theories that investigators may find useful [10,41,50-56].

### Stakeholder priorities and engagement in change

Successful implementation of evidence-based interventions largely depends on their fit with the preferences and priorities of those who shape, deliver, and participate in healthcare. Stakeholders in implementation, and thus in implementation research, include treatment or guideline developers, researchers, administrators, providers, funders, community-based organizations, consumers, families, and perhaps legislators who shape reimbursement policies (see Mendel *et al.*’ article [57] for a framework that outlines different levels of stakeholders). These stakeholders are likely to vary in their knowledge, perceptions, and preferences for healthcare. Their perspectives contribute substantially to the context of implementation and must be understood and addressed if the implementation effort is to succeed. A National Institute of Mental Health Council workgroup report [58] calls for the engagement of multiple stakeholder perspectives, from concept development to implementation, in order to improve the sustainability of evidence-based services in real-world practice. The engagement of key stakeholders in implementation research affects both the impact of proposed implementation efforts, the sustainability of the proposed change, and the feasibility and ultimate success of the proposed research project. Thus, implementation research grant proposals should convey the extent and manner in which key stakeholders are engaged in the project.

Stakeholders and researchers can forge different types of collaborative relationships. Lindamer *et al.*[59] describe three different approaches researchers and stakeholders can take that vary with respect to the level of participation of the stakeholders and community in decisions about the research. In the ‘community-targeted’ approach, stakeholders are involved in recruitment and in the dissemination of the results. In the ‘community-based’ approach, stakeholders participate in the selection of research topics, but the researcher makes the final decision on the study design, methodology, and analysis of data. Finally, the ‘community-driven’ approach or community-based participatory research (CBPR) approach entails participation of the stakeholders in all aspects of the research. Some authors advocate for the CBPR model as a strategy to decrease the gap between research and practice because it addresses some of the barriers to implementation and dissemination [60-62] by enhancing the external validity of the research and promoting the sustainability of the intervention. Kerner *et al.*[62] note:

‘When community-based organizations are involved as full partners in study design, implementation, and evaluation of study findings, these organizations may be more amenable to adopting the approaches identified as being effective, as their tacit knowledge about ‘what works’ would have been evaluated explicitly through research.’

Stakeholder analysis can be carried out to evaluate and understand stakeholders’ interests, interrelations, influences, preferences, and priorities. The information gathered from stakeholder analysis can then be used to develop strategies for collaborating with stakeholders, to facilitate the implementation of decisions or organizational objectives, or to understand the future of policy directions [63,64].

Implementation research grant applications are stronger when preliminary data, qualitative or quantitative, reflect stakeholder preferences around the proposed change. Engagement is also reflected in publications that the principal investigator (PI) and key stakeholders have shared in authorship, or methodological details that reflect stakeholder priorities. Letters of support are a minimal reflection of stakeholder investment in the proposed implementation project.

### Context: Setting’s readiness to adopt new services/ treatments/ programs

Implementation research proposals are strengthened by information that reflects the setting’s readiness, capacity, or appetite for change, specifically around adoption of the proposed evidence-based treatment. This is not to say that all implementation research should be conducted in settings with high appetite for change. Implementation research is often criticized for disproportionate focus on settings that are eager and ready for change. ‘Cherry picking’ sites, where change is virtually guaranteed, or studying implementation only with eager and early adopters, does not produce knowledge that can generalize to usual care, where change is often challenging. The field of implementation science needs information about the process of change where readiness varies, including settings where change is resisted.

Preliminary data on the organizational and policy context and its readiness for change can strengthen an application. Typically viewed as ‘nuisance’ variance to be controlled in efficacy and effectiveness research, contextual factors are key in implementation research [65-67]. The primacy of context is reflected in the choice of ‘it’s all about context’ as a theme at the 2011 NIH Training Institute in Dissemination and Implementation Research in Health [68]. Because organization, policy, and funding context may be among the strongest influences on implementation outcomes, context needs to be examined front and center in implementation research [69]. A number of scales are available to capture one key aspect of context, the setting’s readiness or capacity for change. Weiner *et al.*[70] extensive review focusing on the conceptualization and measurement of organizational readiness for change identified 43 different instruments; though, they acknowledged substantial problems with the reliability and validity of many of the measures. Due in part to issues with reliability and validity of the measures used in the field, work in this area is ongoing [71,72].

Other approaches to assessing readiness have focused on organizational culture, climate, and work attitudes [73], and on providers’ attitudes towards evidence-based practices [21,22,74]. Furthermore, a prospective identification of implementation barriers and facilitators can be helpful in demonstrating readiness to change, increasing reviewers’ confidence that the PI has thoroughly assessed the implementation context, and informing the selection of implementation strategies (discussed in the following section) [75-77]. An evaluation of barriers and facilitators can be conducted through qualitative [78-80] or survey [81,82] methodology. In fact, a number of scales for measuring implementation barriers have been developed [74,83,84]. Letters from agency partners or policy makers, while weaker than data, can also be used to convey the setting’s readiness and capacity for change. Letters are stronger when they address the alignment of the implementation effort to setting or organizational priorities or to current or emergent policies.

### Implementation strategy/process

Though the assessment of implementation barriers can play an important role in implementation research, the ‘rising bar’ in the field demands that investigators move beyond the study of barriers to research that generates knowledge about the implementation processes and strategies that can overcome them. Accordingly, the NIH has prioritized efforts to ‘identify, develop, and refine effective and efficient methods, structures, and strategies to disseminate and implement’ innovations in healthcare [7].

A number of implementation strategies have been identified and discussed in the literature [36,85-87]. However, as the Improved Clinical Effectiveness through Behavioural Research Group notes [38], the most consistent finding from systematic reviews of implementation strategies is that most are effective some, but not all of the time, and produce effect sizes ranging from no effect to a large effect. Our inability to determine how, why, when, and for whom these strategies are effective is hampered in large part by the absence of detailed descriptions of implementation strategies [40], the use of inconsistent language [44], and the lack of clear theoretical justification for the selection of specific strategies [39]. Thus, investigators should take great care in providing detailed descriptions of implementation strategies to be observed or empirically tested. *Implementation Science* has endorsed [40] the use of the WIDER Recommendations to Improve Reporting of the Content of Behaviour Change Interventions [88] as a means of improving the conduct and reporting of implementation research, and these recommendations will undoubtedly be useful to investigators whose proposals employ implementation strategies. Investigators may also find the Standards for Quality Improvement Reporting Excellence (SQUIRE) helpful [89]. Additional design specific reporting guidelines can be found on the Equator Network website [90]. The selection of strategies must be justified conceptually by drawing upon models and frameworks that outline critical implementation elements [10]. Theory should be used to explain the mechanisms through which implementation strategies are proposed to exert their effects [39], and it may be helpful to clarify the proposed mechanisms of change through the development of a logic model and illustrate the model through a figure [91].

According to Brian Mittman, in addition to being theory-based, implementation strategies should be: multifaceted or multilevel (if appropriate); robust or readily adaptable; feasible and acceptable to stakeholders; compelling, saleable, trialable, and observable; sustainable; and scalable [92,93]. We therefore emphasize taking stock of the budget impact of implementation strategies [94] as well as any cost and cost-effectiveness data related to the implementation strategies [95]. Although budget impact is a key concern to administrators and some funding agencies require budget impact analysis, implementation science to date suffers a dearth of economic evaluations from which to draw [96,97].

The empirical evidence for the effectiveness of multifaceted strategies has been mixed, because early research touted the benefits of multifaceted strategies [98,99], while a systematic review of 235 implementation trials by Grimshaw *et al.* found no relationship between the number of component interventions and the effects of multifaceted interventions [100]. However, Wensing *et al.*[101] note that while multifaceted interventions were assumed to address multiple barriers to change, many focus on only one barrier. For example, providing training and consultation is a multifaceted implementation strategy; however, it primarily serves to increase provider knowledge, and does not address other implementation barriers. Thus, Wensing *et al.*[101] argue that multifaceted interventions could be more effective if they address different types of implementation barriers (*e.g.*, provider knowledge and the organizational context). While the methods for tailoring clinical interventions and implementation strategies to local contexts need to be improved [102], intervention mapping [103] and a recently developed ‘behaviour change wheel’ [104] are two promising approaches.

Proposals that employ multifaceted and multilevel strategies that address prospectively identified implementation barriers [102] may be more compelling to review committees, but mounting complex experiments may be beyond the reach of many early-stage investigators and many grant mechanisms. However, it is within the scope of R03, R21, and R34 supported research to develop implementation strategies and to conduct pilot tests of their feasibility and acceptability—work that can strengthen the case for sustainability and scalability. Proposal writers should provide preliminary work for implementation strategies in much the same way that intervention developers do, such as by providing manuals or protocols to guide their use, and methods to gauge their fidelity. Such work is illustrated in the pilot study conducted by Kauth *et al.*[105], which demonstrated that an external facilitation strategy intended to increase the use of cognitive behavioral therapy within Veteran Affairs clinics was a promising and low-cost strategy; such pilot data would likely bolster reviewers’ confidence that the strategy is feasible, scalable, and ultimately, sustainable. Investigators should also make plans to document any modifications to the intervention and, if possible, incorporate adaptation models to the implementation process, because interventions are rarely implemented without being modified [67,106].

While providing detailed specification of theory-based implementation strategies is critical, it is also imperative that investigators acknowledge the complexity of implementation processes. Aarons and Palinkas [107] comment:

‘It is unrealistic to assume that implementation is a simple process, that one can identify all of the salient concerns, be completely prepared, and then implement effectively without adjustments. It is becoming increasingly clear that being prepared to implement EBP means being prepared to evaluate, adjust, and adapt in a continuing process that includes give and take between intervention developers, service system researchers, organizations, providers, and consumers.’

Ultimately, proposals that reflect the PI’s understanding of the complexity of the process of implementing evidence-based practices and that provide supporting detail about strategies and processes will be perceived as more feasible to complete through the proposed methods.

### Team experience with the setting, treatment, implementation process, and research environment

Grant reviewers are asked to specifically assess a PI’s capacity to successfully complete a proposed study. Grant applications that convey the team’s experience with the study setting, the treatment whose implementation is being studied, and implementation processes help convey capacity and feasibility to complete an implementation research project [108].

The reader should observe that NIH gives different scores for the team experience with the setting and for the research environment (http://grants.nih.gov/grants/writing_application.htm) but the purpose of both sections is demonstrating capacity to successfully carry out the study as proposed. Investigators can convey capacity through a variety of ways. Chief among them is building a strong research team, whose members bring depth and experience in areas the PI does not yet have. Implementation research exemplifies multidisciplinary team science, informed by a diverse range of substantive and methodological fields [96,109]. A team that brings the needed disciplines and skill sets directly to the project enhances the project’s likelihood of success. Early-stage implementation researchers who collaborate or partner with senior investigators reassure reviewers that the proposed work will benefit from the senior team member’s experience and expertise. Similarly, collaborators play important roles in complementing, or rounding out, the PI’s disciplinary perspective and methodological skill set. Early career investigators, therefore, should surround themselves with more established colleagues who bring knowledge and experience in areas key to the study aims and methods. The narrative should cite team members’ relevant work, and their prior work can be addressed in a discussion of preliminary studies. Additionally, the new formats for NIH biosketches and budget justifications enable a clear portrayal of what each team member brings to the proposed study.

For the NIH applications, the research environment is detailed in the resources and environment section of a grant application. Here, an investigator can describe the setting’s track record in implementation research; research centers, labs, and offices that the PI can draw on; and structural and historic ties to healthcare settings. For example, a PI can describe how their project will draw upon the University’s CTSA program [110], statistics or design labs, established pools of research staff, and health services research centers. Preliminary studies and biosketches provide additional ways to convey the strengths of the environment and context within which an investigator will launch a proposed study.

In summary, researchers need to detail the strengths of the research environment, emphasizing in particular the resources, senior investigators, and research infrastructure that can contribute to the success of the proposed study. A strong research environment is especially important for implementation research, which is typically team-based, requires expertise of multiple disciplines, and requires strong relationships between researchers and community based health settings. Investigators who are surrounded by experienced implementation researchers, working in a setting with strong community ties, and drawing on experienced research staff can inspire greater confidence in the proposed study’s likelihood of success.

### Feasibility of proposed research design and methods

One of the most important functions of preliminary work is to demonstrate the feasibility of the proposed research design and methods. Landsverk [108] urges PIs to consider every possible question reviewers might raise, and to explicitly address those issues in the application. Data from small feasibility studies or pilot work around referral flow; participant entry into the study; participant retention; and the extent to which key measures are understood by participants, acceptable for use, and capture variability can demonstrate that the proposed methods are likely to work. The methods section should contain as much detail as possible, as well as lay out possible choice junctures and contingencies, should methods not work as planned. It is not only important to justify methodological choices, but also to discuss why potential alternatives were not selected. For example, if randomization is not feasible or acceptable to stakeholders, investigators should make that clear. Letters from study site collaborators can support, but should not replace, the narrative’s detail on study methods. For example, letters attesting the willingness of study sites to be randomized or to support recruitment for the proposed timeframe can help offset reviewer concerns about some of the real-world challenges of launching implementation studies.

### Measurement and analysis

A grant application must specify a measurement plan for each construct in the study’s overarching conceptual model or guiding theory, whether those constructs pertain to implementation strategies, the context of implementation, stakeholder preferences and priorities, and implementation outcomes [111]. Yet, crafting the study approach section is complicated by the current lack of consensus on methodological approaches to the study of implementation processes, measuring implementation context and outcomes, and testing implementation strategies [112,113]. Measurement is a particularly important aspect of study methods, because it determines the quality of data. Unlike efficacy and effectiveness studies, implementation research often involves some customization of an intervention to fit local context; accordingly, measurement plans need to address the intervention’s degree of customization versus fidelity [97]. Moreover, implementation science encompasses a broad range of constructs, from a variety of disciplines, with little standardization of measures or agreement on definitions of constructs across different studies, fields, authors, or research groups, further compounding the burden to present a clear and robust measurement plan along with its rationale. Two current initiatives seek to advance the harmonization, standardization, and rigor of measurement in implementation science, the U.S. National Cancer Institute’s (NCI) Grid-Enabled Measures (GEM) portal [114] and the Comprehensive Review of Dissemination and Implementation Science Instruments efforts supported by the Seattle Implementation Research Conference (SIRC) at the University of Washington [115]. Both initiatives engage the implementation science research community to enhance the quality and harmonization of measures. Their respective web sites are being populated with measures and ratings, affording grant writers an invaluable resource in addressing a key methodological challenge.

Key challenges in crafting the analysis plan for implementation studies include: determining the unit of analysis, given the ‘action’ at individual, team, organizational, and policy environments; shaping meditational analyses given the role of contextual variables; and developing and using appropriate methods for characterizing the speed, quality, and degree of implementation. The proposed study’s design, assessment tools, analytic strategies, and analytic tools must address these challenges in some manner [113]. Grant applications that propose the testing of implementation strategies or processes often provide preliminary data from small-scale pilot studies to examine feasibility and assess sources of variation. However, the magnitude of effects in small pilots should be determined by clinical relevance [113], given the uncertainty of power calculations from small scale studies [116].

### Policy/funding environment; leverage or support for sustaining change

PIs should ensure that grant applications reflect their understanding of the policy and funding context of the implementation effort. Health policies differ in many ways that impact quality [117], and legal, reimbursement, and regulatory factors affect the adoption and sustainability of evidence-based treatments [118]. Raghavan *et al.*[119] discuss the policy ecology of implementation, and emphasize that greater attention should be paid to marginal costs associated with implementing evidence-based treatments, including expenses for provider training, supervision, and consultation. Glasgow *et al.*[120] recently extended their heretofore behaviorally focused RE-AIM framework for public health interventions to health policies, revealing the challenges associated with policy as a practice-change lever.

PIs can address the policy context of the implementation initiative through the narrative, background literature, letters of support, and the resource and environment section. Proposals that address how the implementation initiative aligns with policy trends enhance their likelihood of being viewed as having high public health significance, as well as greater practical impact, feasibility, and sustainability. It is important to note that it may behoove investigators to address the policy context within a proposal even if it is not likely to be facilitative of implementation, because it demonstrates to reviewers that the investigator is not naïve to the challenges and barriers that exist at this level.

## Summary

We identify and discuss ten key ingredients in implementation research grant proposals. The paper reflects the team’s experience and expertise: writing for federal funding agencies in the United States. We acknowledge that this will be a strength for some readers and a limitation for international readers, whom we encourage to contribute additional perspectives. Setting the stage with careful background detail and preliminary data may be more important for implementation research, which poses a unique set of challenges that investigators should anticipate and demonstrate their capacity to manage. Data to set the stage for implementation research may be collected by the study team through preliminary, feasibility, or pilot studies, or the team may draw on others’ work, citing background literature to establish readiness for the proposed research.

Every PI struggles with the challenge of fitting into a page-limited application the research background, methodological detail, and information that can convey the project’s feasibility and likelihood of success. The relative emphasis on, and thus length of text addressing, the various sections of a grant proposal varies with the program mechanism, application ‘call,’ and funding source. For NIH applications, most attention and detail should be allocated to the study method because the ‘approach’ section is typically weighted most heavily in scoring. Moreover, the under-specification or lack of detail in study methodology usually receives the bulk of reviewer criticism. Well-constructed, parsimonious tables, logic models, and figures reflecting key concepts and the analytic plan for testing their relationships all help add clarity, focus reviewers, and prevent misperceptions. All implementation research grants need to propose aims, study questions, or hypotheses whose answers will advance implementation science. Beyond this fundamental grounding, proposed implementation studies should address most, if not all, of the ingredients identified here. While no application can include a high level of detail about every ingredient, addressing these components can help assure reviewers of the significance, feasibility, and impact of the proposed research.

## Endnotes

^a^For more information regarding different grant mechanisms, please see: http://grants.nih.gov/grants/funding/funding_program.htm.

## Competing interests

The authors declare that they have no competing interests.

## Authors’ contributions

EKP conceived the idea for this paper and led the writing. BJP, AAB, AMH, and RLS contributed to the conceptualization, literature review, and the writing of this manuscript. All authors read and approved the final manuscript.

## Authors’ information

EKP directs the Center for Mental Health Services Research at Washington University in St. Louis (NIMH P30 MH085979), the Dissemination and Implementation Research Core (DIRC) of the Washington University Institute of Clinical and Translational Sciences (NCRR UL1RR024992), and the Implementation Research Institute (NIMH R25 MH080916).
